# Two-stage flux balance analysis of metabolic networks for drug target identification

**DOI:** 10.1186/1752-0509-5-S1-S11

**Published:** 2011-06-20

**Authors:** Zhenping Li, Rui-Sheng Wang, Xiang-Sun Zhang

**Affiliations:** 1 School of Information, Beijing Wuzi University, Beijing 101149, China; 2 Department of Physics, Pennsylvania State University, University Park, PA 16802, USA; 3 Academy of Mathematics and Systems Science, CAS, Beijing 100190, China

## Abstract

**Background:**

Efficient identification of drug targets is one of major challenges for drug discovery and drug development. Traditional approaches to drug target identification include literature search-based target prioritization and *in vitro* binding assays which are both time-consuming and labor intensive. Computational integration of different knowledge sources is a more effective alternative. Wealth of omics data generated from genomic, proteomic and metabolomic techniques changes the way researchers view drug targets and provides unprecedent opportunities for drug target identification.

**Results:**

In this paper, we develop a method based on flux balance analysis (FBA) of metabolic networks to identify potential drug targets. This method consists of two linear programming (LP) models, which first finds the steady optimal fluxes of reactions and the mass flows of metabolites in the pathologic state and then determines the fluxes and mass flows in the medication state with the minimal side effect caused by the medication. Drug targets are identified by comparing the fluxes of reactions in both states and examining the change of reaction fluxes. We give an illustrative example to show that the drug target identification problem can be solved effectively by our method, then apply it to a hyperuricemia-related purine metabolic pathway. Known drug targets for hyperuricemia are correctly identified by our two-stage FBA method, and the side effects of these targets are also taken into account. A number of other promising drug targets are found to be both effective and safe.

**Conclusions:**

Our method is an efficient procedure for drug target identification through flux balance analysis of large-scale metabolic networks. It can generate testable predictions, provide insights into drug action mechanisms and guide experimental design of drug discovery.

## Background

Drug target is a key molecule involved in a particular metabolic or signaling pathway that is specific to a disease condition or the survival of a microbial pathogen [1,2]. Identification and validation of drug target is the essential first step in new drug discovery and development. Drugs can be designed to modify the functioning of the pathway in the diseased state by inhibiting a key molecule, or to enhance the normal pathway by promoting specific molecules that may have been affected in the diseased state. For the diseases caused by microbial pathogen, drugs usually are designed to inhibit the essential components of the pathogen to disrupt its survival. In all the cases, drugs should be designed in such a way as not to affect any other important molecules, since modification of non-disease-causing molecules may lead to undesirable side effects [3].

In pharmaceutics, drugs generally fail in the clinic for two reasons: they either do not work or are proved to be unsafe [1]. For example, if components other than disease-causing compounds are affected by a drug, toxicity or side effect will arise; on the other hand, if disease-causing compounds are not inhibited by a drug, then lack of efficacy will arise. Both of these problems have been attributed to sloppy early target discovery and are among the main challenges in developing new drugs. Traditional drug development approaches focused more on the efficacy of drugs than their toxicity, which does not meet the increasing demand of public health on new drug development. On the other hand, recent drug research in post-genomic era stresses on the identification of specific biological targets such as enzymes or proteins for drugs, which can be manipulated to produce the desired effect of curing a disease with minimum disruptive side effects [1,4]. With the complete sequencing of human and bacterial genomes and the subsequent accumulation of genomic, proteomic, and metabolomic data, systems biology approaches or network-based analyses hold great promise for identifying drug targets by utilizing biological networks, such as gene regulatory networks, metabolic networks and protein interaction networks [5-16]. Among these methods, one class is to identify drug targets by analyzing the topological feature of protein interaction networks or metabolic networks [6,8,9,17]. For example, Guimerà et al. proposed a module-based approach to characterize the roles of enzymes according to the modular structure of metabolic networks, which is promising for identification of drug targets [6]. Hormozdiari et al. proposed sparest cut strategies to identify potential multiple-drug targets in pathogenic protein-protein interaction networks with goal of disrupting known essential pathways or complexes in pathogens [8]. In addition, flux balance analysis (FBA) of genome-scale metabolic networks is another important class of methods for drug target identification. Usually methods in this category aim to predict essential enzymes which are critical to the survival and growth of pathogens [15,18-21]. Raman et al. constructed a comprehensive model of mycolic acid synthesis metabolic pathway in the pathogen *Mycobacterium tuberculosis* and used FBA to do *in silico* systematic gene deletions which identify proteins essential for this pathway and lead to identification of anti-tubercular drug targets [18]. *Plasmodium falciparum* is the primary agent of the best-known tropical disease malaria. Plata et al. reconstructed a genome-scale metabolic network of P. *falciparum* and did FBA for simulating gene deletion [20]. Their model reproduced the phenotypes of experimental gene knockouts and drug inhibition assays with high accuracy and identified 40 essential genes as enzymatic drug targets. Recently, a few studies have been done on prediction of drug-target interaction by integration of chemical, genomic and pharmacological data [11-13,22] . In short, wealth of various types of omics data are changing the way researchers view drug targets and provides unprecedent opportunities for drug target identification.

For pathogenic diseases, drugs are designed to act on the pathogen directly, and drug targets are those enzymes crucial for the survival and growth of the pathogen, which can be identified by FBA-based growth simulation or sparse cut strategies [8,18,20]. The pathogenic diseases are cured by inhibiting essential enzymes (drug targets) using drugs. For nonpathogenic diseases, drugs act on human enzymes and adjust the reactions catalyzed by these enzymes to make metabolism normal and cure the diseases caused by metabolic disorders [7,14]. Although many methods have been developed for drug target identification, most of them do not consider the factor of side effects, which may be the main reason why only modest results have been obtained so far. Recently, a new drug target identification model based on metabolic networks has been proposed by Sridhar et al. [23,24], in which a set of enzymes (drug targets) is to be found to inhibit disease-causing compounds through drugs’ action on these enzymes and meanwhile reduce the side effects caused to non-disease-causing compounds as much as possible. In other words, inhibition of the identified drug targets will stop the production of a given set of disease-causing compounds, and meanwhile eliminate a minimum number of non-disease-causing compounds. In their models, the side effect of a drug is defined as the number of non-disease-causing compounds eliminated while drugs inhibit the disease-causing compounds. They presented a scalable heuristic iterative algorithm as well as a branch-and-bound exact algorithm for solving the formulated drug target identification problem [23,24]. Song et al. developed a double iterative optimization algorithm for the same problem [25]. Li et al. further formulated this metabolic network-based drug target identification model as an integer linear programming (ILP) which ensures that optimal solutions can be exactly and efficiently obtained without any heuristic manipulation [26]. Instead of using flux balance analysis, the drug target discovery model in this class is based on the logic biochemical relationships between reactions, enzymes and compounds: a reaction is inhibited if and only if at least one of its reactant metabolites is inhibited, and a product metabolite is inhibited if and only if all reactions producing this metabolite are inhibited [27]. Aiming at minimizing the side effects of drug targets, this model does not need to determine biological objective functions for optimizing flux distribution.

The drug target identification model mentioned above is qualitative and explores properties of metabolic networks from a topological view. However, although the definition of damage in such a model reflects side effects to some extent, it is still too coarse and cannot capture the quantitative relationships among reactions, metabolites and enzymes. In the process of metabolism, the mass flows of metabolites and the fluxes of reactions satisfy balance relationships. If disease-causing compounds are completely inhibited by manipulating drug targets, some non-disease-causing compounds may also be eliminated, which may change the concentration or mass flow of some other non-disease-causing compounds. If the changed concentration or mass flow of these non-disease-causing compounds is out of a healthy range, some symptoms of side effects will appear. In fact, although the accumulation of disease-causing compounds in a sophisticated metabolic system may result in diseases, it is not reasonable to inhibit them completely. We only need to adjust their concentration or mass flow to a healthy range by certain medication strategies. For example, the healthy range of normal empty blood sugar concentration of a person is [0, 6.11] mmol/L. If his empty blood sugar concentration is larger than 7.0 mmol/L, then he may be diagnosed to be a diabetes patient. To cure diabetes, we need to reduce their empty blood sugar concentration to a healthy range. Sridhar et al.’s qualitative drug target identification model cannot handle this case. In [7], Vera et al. proposed a method called optimization program for drug discovery (OPDD) to identify enzyme targets in enzymopathies by integration of metabolic models and biomedical data. An existing S-system model and literature information about the human hyperuricemia were used to detect single-enzyme targets and two-enzyme targets. This method needs to solve a large number of optimization programs and select the most feasible solution by additional criteria. Furthermore, side effects are not taken into account. In this paper, we propose a method to identify drug target based on flux balance analysis (FBA), in which we consider a quantitative and more reasonable definition of damage to reflect side effects of drug action, that is, the deviation of the mass flow of non-disease-causing metabolites from their health range. Our method consists of two linear programming models: one is to find the optimal fluxes of reactions and the mass flows of metabolites in the pathologic state, and the other is to determine the fluxes and mass flows in the medication state with the minimal side effect caused by the medication. Then drug targets are identified by comparing the fluxes of reactions in both states and checking the reactions whose fluxes are changed. An illustrative example is given to show that the drug target identification problem can be solved effectively by our method. We also apply our method to a hyperuricemia-related purine metabolic pathway. Known drug targets for hyperuricemia are correctly identified by our two-stage FBA method, and the side effects of these targets are also taken into account. A number of other promising drug targets are found to be both effective and safe.

## Methods

Metabolism, which comprises the complete set of biochemical reactions in a living cell, is one of the most complex cellular processes. Metabolic networks connect biochemical reactions via substrate and product substances called metabolites. In a metabolic network, enzymes catalyze reactions which take substrates and produce metabolites. Such processes constitute the whole metabolism system of a living organism. However, the malfunctions of some enzymes may lead to production of excessive concentration or mass flow of certain compounds in a sophisticated metabolic system, and thereby may result in diseases [28]. Such compounds are generally considered as disease-causing compounds because they are directly relevant to the diseases. The remaining compounds in the metabolic system are all considered as non-disease-causing compounds. On the other hand, those enzymes are considered as drug targets, if manipulating them by drugs the concentration or mass flow of disease-causing compounds can be adjusted to a healthy range. Hence, the drug target identification problem is to identify such an enzyme set that can be manipulated by drugs to adjust the mass flow of all disease-causing compounds to a healthy range, and meanwhile reduce the gap between the mass flow of non-disease-causing compounds after medication and their healthy range as much as possible. The sum of gaps between the mass flow of all non-disease-causing compounds and their healthy state range is defined as the side effects (of the drug targets).

### Metabolic network representation

A metabolic network is generally a biochemical network, in which chemical compounds and metabolites are represented by nodes and reactions catalyzed by one or several certain enzymes are denoted by directed edges. In order to make drug target identification easily understood, we use another graphical representation of metabolic networks [29], in which a metabolic network is built up of substrates that are connected to one another not through single links, but through physical entities denoting reactions (enzymes). A metabolic network in this type of representation is a directed bipartite graph and has two types of nodes. One type represents chemical reactions and the other metabolites. A directed edge from a reaction to a metabolite means that the metabolite is a product of the reaction. A directed edge from a metabolite to a reaction represents that the metabolite is a reactant of the reaction. A reversible reaction is considered as two separate reactions corresponding to forward and backward reactions. This representation allows us conveniently to express the relations between substrates, reactions and products by the topology of metabolic networks.

Suppose that there are m metabolites {*C_1_*, *C*_2_, …,*C_m_*} and *n* reactions {*R*_1_, *R_2_*, …, *R_n_*} in a metabolic network. *S* = [*s_i_*_,_*_j_*]*_m_*_×_*_n_* and *T* = [*t_j_*_,_*_i_*]*_n_*_×_*_m_* are the stoichiometric coefficient matrices of reactions. The *k*th column of matrix *S* denotes the coefficients of reactants in reaction *R_k_*, while the *k*th row of matrix *T* denotes the coefficients of metabolites produced by reaction *R_k_*. We can obtain the *k*th column of matrix *S* and the *k*th row of matrix *T* from the chemical equation of reaction *R_k_ .* Conversely, the chemical equation of reaction *R_k_* can be deduced from the *k*th column of matrix *S* and the *k*th row of matrix *T.* For example, the chemical equation of reaction *R_k_* is

2*C*_1_ + 3*C*_2_*→C_5_*+ 2*C*_6_

Then *s*_l,_*_k_* = 2, *s*_2,_*_k_* = 3, *s_i_*_,_*_k_* = 0,*i* ≠ 1, 2 and *t_k_*_,5_ = 1, *t_k_*_,6_ = 2, *t_k_*_,_*_j_* = 0, *j* ≠ 5,6.

**Figure 1:**
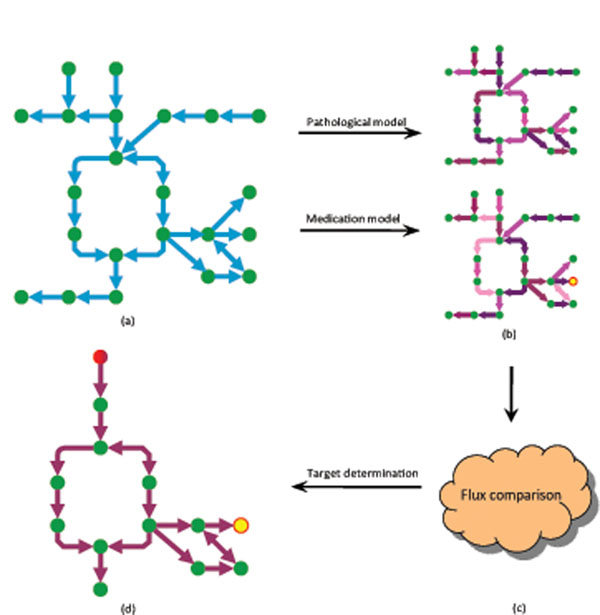
**General scheme of the two-stage flux balance analysis (FBA) method for drug target identification.** Given a metabolic pathway related to a disease (a), our two-stage FBA method first calculates optimal fluxes of reactions and mass flows of metabolites in the pathologic state (b). Then, assuming some medication strategy can adjust the abnormal level of the disease-causing compound (the node marked in yellow), the two-stage FBA method determines the fluxes of reactions and the mass flows of metabolites with minimum side effects in the medication state (b). Different colors of the edges in (b) represent different fluxes. By comparing the reaction fluxes in the pathologic state and medication state (c), a sub-network is constructed and potential drug targets (the node marked in red) are identified (d).

After formulating a metabolic network into a bipartite digraph as described above, our method for drug target identification, called as two-stage FBA method, is formulated into two linear programming models. The general scheme is shown in Figure 1, which we will introduce step by step in the following subsections.

### Determining pathologic metabolite mass flows and reaction fluxes

Kinetic modeling of large-scale metabolic networks is often impossible due to lack of specific enzyme rate data. A simple and useful alternative for analysis of metabolic capabilities of cellular systems is flux balance analysis (FBA) [30]. FBA ignores metabolite mass flow, enzyme activity and transient dynamics and focuses on stoichiometry of metabolic reactions, mass balance and steady state, which makes it able to analyze large-scale metabolic systems in complex organisms [27]. In FBA models, the concentration change of each species over time follows mass balance and can be defined in terms of the flux (reaction rate) and stoichiometry of each reaction in which the species participates. The transient mass balance can be further simplified to only consider the steady state, which leads to **S** · **v** = 0, where **v** = (*v_1_,v_2_*, … ,*v_n_*)*^T^* denotes the flux vector of the reactions in the stoichiometric matrix **S** = *-S* + *T^T^.* In metabolic systems, the feasible region in the steady-state flux space may be too large to be meaningful. FBA overcomes this by adding biologically meaningful objectives into the model, such as maximization of growth rate, maximization of ATP production, and minimization of nutrient uptake. These objectives can be represented by a linear combination of reaction fluxes of interest, which results in a linear programming model:

max **c***^T^ ·***v** (1)

s.t. **S** · **v** = 0 (2)

**V**_min_ ≤ **v** ≤ **v**_max_ (3)

where **c** in the objective function (1) is a vector of weights for the fluxes **v**, (2) is the set of mass balance constraints, and (3) is the set of enzymatic capacity constraints. FBA has been widely used for *in silico* phenotype prediction. For more methodological details of FBA, one can refer to [31,32].

Given a metabolic network in the pathologic state, we delete the reactions that cannot take place because its catalyzing enzyme is inhibited in the disease state. Although the metabolic network is in the pathologic state, it still can produce as much biomass or energy (e.g. ATP) as possible so as to maintain tissue growth. So we can determine the flux of each reaction and the mass flow of each metabolite in the pathologic state by a FBA optimization model. Let *v_j_* denote the flux of reaction *R_j_*, *x*_i_ denote the mass flow of metabolite *C_i_*_,_ that is, the mass flow of metabolite *C_i_* produced (or consumed) by all the reactions it involves in the metabolic network. We use the following linear programming model to determine the mass flows of metabolites and the fluxes of reactions in the pathologic state:(4)(5)(6)(7)

0 ≤ *v_j_* ≤ *U_j_*_,_*j* = 1,2, … , *n* (8)

0 ≤ *x_i_* ≤ *q_i_*_,_*_i_* = 1,2, … , *m* (9)

The objective function denotes the maximization of mass flows of certain metabolites. For example, if we want to maximize the mass flows of metabolites in the biomass reaction, we can set *c*_biomass,_*_i_* = 1 and *C_j_*_,_*_i_*= 0, *j* ≠ biomass. Eq. (5) is the mass balance constraint of each intermediate metabolite. Constraint (6) defines that the mass flow of each metabolite is equal to the weighted sum of the fluxes of all reactions (if any) that consume this metabolite. Similarly, constraint (7) guarantees that the mass flow of each metabolite is equal to the weighted sum of the fluxes of all reactions (if any) that produce this metabolite. Constraints (14) and (15) represent the capacity limits of reaction flux and metabolite mass flow in the pathologic state, where *U_j_* and *q_i_* are the upper bounds of variables *v_j_* and *x_i_* respectively.

### Determining medication metabolite mass flows and reaction fluxes

In the pathologic state, the mass flows of some metabolites are out of healthy ranges which directly result in the disease symptoms. For example, if the healthy range of the *j*th metabolite’s mass flow is [*a_i_* ,*b_j_*], it means that *x_j_* should satisfy *a_j_* ≤ *x_j_* ≤ *b_j_ .* If *x_j_* >*b_j_* or *x_j_* <*a_j_*, some fluxes of biochemical reactions should be adjusted by using drugs so that *x_j_* ∈ [*a_j_*, *b_j_*]*.* At this time, the metabolite *C_j_* is the disease-causing compound, other metabolites are non-disease-causing compounds, and the biochemical reactions whose fluxes are to be enzymatically adjusted by using drugs are viewed as drug targets. In this adjustment process, the mass flows of some other non-disease-causing compounds may change to be out of their health ranges, which we define as the side effects of the drugs. A good drug should be potent and have minimal side effects. Aiming to minimize the side effects, we can find the mass flows of metabolites and the fluxes of reactions in the medication state by using the following linear programming model:(10)(11)(12)(13)

0 ≤ *v_j_* ≤ *U_j_*_,_*j* = 1,2, … ,*n* (14)

*a*_j_ ≤ *x_i_* ≤ *b_i_*, *i* ∈ *P* (15)(14)

where *N* is the set of non-disease-causing compounds and *P* is the set of disease-causing compounds, *a_i_ b_i_*are respectively the healthy lower and upper bounds of the mass flow of the target compound *C_i_* and  are variables representing the deviation of the mass flow of *C_i_* from its healthy range. Constraint (11) is only for intermediate metabolites. Constraint (12) is for intermediate metabolites and those metabolites that are only consumed (not produced) in the system. Constraint (13) is for intermediate metabolites and those metabolites that are only produced (not consumed) in the system.

### Identifying drug targets and drug dose for diseases

After we obtain the flux vector of reactions **v**^0^ in the pathologic state and the flux vector of reactions **v**^1^ in the medication state, by comparing **v**^0^ and **v**^1^, we can easily find the reactions whose flux has been changed by medication. We construct a sub-metabolic network by using all these reactions whose fluxes have been changed by medication along with their reactants and products. All the compounds with zero in-degree are then deleted, that is, delete all the compounds which is not a product of any reaction in this subnetwork. These compounds come into the metabolism process from the outside of the system. In the sub-metabolic network consisting of changed reactions and their related compounds, all the reactions that have no reactants are identified. These reactions (equivalently, enzymes catalyzing these reactions are actually the boundary or source of the system and determined as drug targets. This indicates that manipulating the concentration of enzymes that catalyze these reactions by drugs can adjust the reaction fluxes so that the mass flows of disease-causing compounds are changed back to the healthy ranges following the paths in the sub-metabolic network. This alignment strategy for finding drug targets is reasonable. For example, Chu and Chen constructed protein-protein interaction networks involved in the apoptosis of cancerous and normal cells to determine cancer-perturbed protein-protein interactions which allows identification of potential apoptosis drug targets for anti-cancer drugs [33].

In [34], it has been indicated that the flux of a reaction is correlated with the concentration level of the enzymes catalyzing this reaction. The concentration of enzymes can be controlled by drugs, so drug dose can be determined according to the flux change of reactions between the pathologic and medication states. Primary experimental methods can also determine the suitable dose to cure the disease.

## Results

In this section, we use an illustrative simulated metabolic network and a real human metabolic pathway to test the effectiveness of our method in detecting potential drug targets. The algorithm is coded by Python script and the LP models are solved by GLPK linear programming/MIP solver GLPSOL.

### An illustrative simulated metabolic network

**Figure 2:**
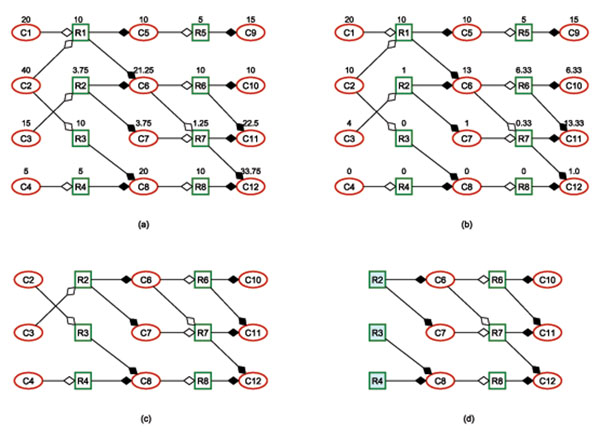
**A simulated metabolic network.** (a) The fluxes of reactions and the mass flows of metabolites in the pathologic state. (b) The fluxes of reactions and the mass flows of metabolites in the medication state. (c) The sub-metabolic network obtained by comparing the reaction fluxes in the pathologic state and medication state. (d) The reactions R2, R3, and R4 corresponding to potential drug targets.

Figure 2(a) is a simulated metabolic network of 12 metabolites and 8 reactions, which can be expressed by the following chemical reaction equations.

R1 : 2C1 + C2→C5 + C6

R2 : 4 C3 → 3 C6 + C7

R3 : 3 C2 → C8

R4 : C4 → 2 C8

R5 : 2 C5 → 3 C9

R6: 2C6→C10 + 2C11

R7: C6 + 3C6→2C11 + 3C12

R8 : 2 C8 → 3 C12

We assume that metabolites C8, C9, C11, C12 are involved in the biomass reaction. In the pathologic state, the upper bounds of all reaction fluxes are taken as 10, and the upper bounds of mass flow of all metabolites are taken as infinity. Solving the pathologic linear programming model, we can obtain the results expressed in Figure 2(a), where the optimal mass flow vector of metabolites is **x**^0^ = (20, 40, 15, 5, 10, 21.25, 3.75, 20,15, 10, 22.5, 33.75) and the optimal flux vector of reactions is **v**^0^ = (10, 3.75,10,5, 5,10, 1.25,10), depicted beside the corresponding nodes. Suppose that the healthy ranges of metabolites C1-C8 are very high, the healthy ranges of other metabolites are 10 ≤ *x*_C9_,*x*_C10_ , *x*_C11_ ≤ 15 and 0 ≤ *x*_C12_ < 1, and the flux upper bound of each reaction is *U_j_* = 10, *j* = 1,2, … , 8. It is easy to see that the disease-causing metabolites are C11 and C12, whose mass flows are out of their health ranges. We can further find the optimal fluxes of reactions and the mass flows of metabolites in the medication state. The optimal mass flow vector of metabolites is **x**^1^ = (20, 10, 4, 0, 10, 13, 1, 0, 15, 6.33, 13.33, 1) and the optimal flux vector of reactions is **v**^1^ = (10, 1, 0, 0, 5, 6.33, 0.33, 0), both shown in Figure 2(b). The side effect is 3.667 since the mass flow of metabolite C10 is 6.33, out of its healthy range 10 ≤ *x*_C10_ ≤ 15.

By comparing **v**^0^ and **v**^1^, we construct a sub-metabolic network, shown in Figure 2(c). According to the method described in the Methods section, potential drug targets are the enzymes which catalyze R2, R3, R4 respectively. If we adjust the fluxes of R2, R3, R4 respectively to be 1, 0, 0 by using drugs, then the mass flows of disease-causing compounds will be in healthy ranges with side effect 3.667. This result can also be obtained by the pathologic model with the flux constraints of reaction R2, R3, and R4 being 1,0,0 respectively. If the healthy ranges of metabolite mass flows are modified to be 10 ≤ *x*_C9_, *x*_C12_ ≤ 15, 5 ≤ *x*_C10_ ≤ 15, and 15 ≤ *x*_C11_ ≤ 20, then the optimal drug targets are still R2, R3, R4, and the side effect is 0, which means that the disease can be cured by medication treatment on the enzymes catalyzing R2, R3, and R4 without causing side effects.

### Detection of drug targets for human hyperuricemia

Hyperuricemia is an enzymopathy which is characterized by the abnormally high level of uric acid in the blood. One of main causes of human hyperuricemia is the increased production of uric acid that results from high levels of purine in the diet and increased purine metabolism. Foods high in the purine, adenine and hypoxanthine are very potent in exacerbating hyperuricemia [35]. From a metabolism view, the main cause of this disease is a functional defect in the enzyme phosphoribosylpy-rophosphate (PRPP) synthetase that controls the synthesis of purine. This defect provokes an increase in its enzymatic activity and leads to an augmentation of degradative metabolic fluxes yielding more uric acid than usual [7,36,37]. The uric acid is stored in the form of urate crystals in some tissues (e.g. joints) and results in the symptoms of hyperuricemia such as acute episodes of arthritic pain and nephropathy. Currently, there are two kinds of medications most often used to treat hyperuricemia: xanthine oxidase inhibitors and uricosurics. Xanthine oxidase inhibitors decrease the production of uric acid by interfering with xanthine oxidase. Allopurinol is one of specific inhibitors of xanthine oxidase that can lead to a drastic reduction in the concentrations of uric acid [38]. Uricosurics increase the excretion of uric acid by reducing the reabsorption of uric acid once the kidneys have filtered it out of the blood. Other treatments of this disease include a symptomatic treatment for joint pain and a restricted diet that precludes consumption of food with high concentrations of purine precursors [7].

**Figure 3:**
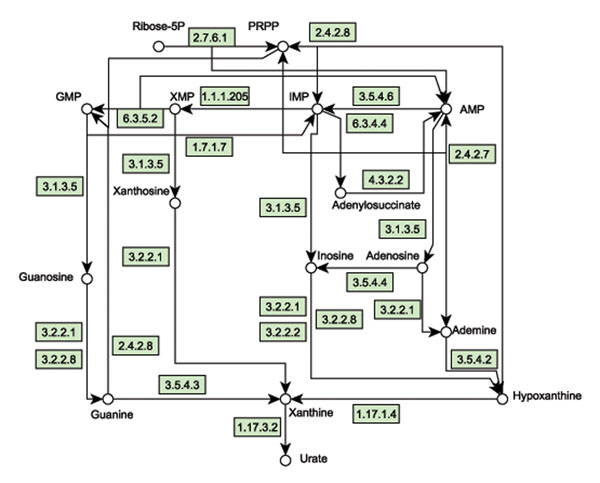
**The human hyperuricemia metabolic pathway.** Only the chemical transformations of main metabolites are shown here. The corresponding detailed reactions can be found in Additional file 1. In this figure, metabolites are represented by small circles. Reactions are the directed edges. Enzymes catalyzing reactions are represented by rectangles and put on the corresponding edges.

Hyperuricemia has been widely investigated and its functional mechanism and metabolic basis is well understood, which makes it a good case study for drug target identification. In this work, we examine whether our method is able to detect enzymes which may be helpful for adjusting the level of uric acid and act as potential drug targets. Since hyperuricemia is closely related to human purine metabolism, we construct a hyperuricemia metabolic pathway by referring to the *Homo sapiens* purine metabolism in KEGG and the purine metabolic model in [7]. Those reactions and metabolites that are obviously irrelevant to the production of uric acid (urate) are not included. The constructed hyperuricemia-related purine metabolic pathway has 23 reactions and 35 compounds, with a number of enzymes catalyzing the reactions. The chemical transformations of main metabolites are shown in Figure 3. This network includes the synthesis, recovery and degradation of purine nucleotides, and the regulation of the enzyme activity for metabolites, either substrates or products. The complete lists of reactions, compounds and enzymes in this pathway are in Additional file 1, Additional file 2, and Additional file 3 respectively.

Since it is known that hyperuricemia is characterized by the abnormally high level of uric acid, we simulate the pathologic state of the metabolic pathway by maximizing the production of uric acid. All the reactions have an identical upper bound of fluxes 10 except for the reaction producing uric acid and the reaction synthesizing purine. As described in Methods section, the pathologic model has a mass balance constraint for each nontrivial intermediate metabolite which is a linear equation of reaction fluxes, and the mass flow of each metabolite is the weighted sum of all fluxes of the reactions producing (or consuming) it. The upper bounds of reaction fluxes and the mass balance constraints together determine the ranges of the mass flows of metabolites, so here we do not set particular bounds for metabolites. Solving this pathologic model for the hyperuricemia metabolic pathway, we find that the abnormal level of uric acid obtained by the pathologic model is 20, and 16 reactions have non-zero fluxes, including the reactions catalyzed by xanthine oxidase, phosphoribosylpy-rophosphate synthetase, hypoxanthine phosphoribosyltransferase, IMP dehydrogenase, AMP deaminase, adenine deaminase, 5’-nucleotidase, etc. The reaction synthesizing PRPP has the highest flux, which is consistent with the fact that functional defect in the enzyme phosphoribosylpy-rophosphate (PRPP) synthetase causes hyperuricemia.

Healthy level of uric acid should be much less than that in hyperuricemia, so we assume that the healthy range of the mass flow of uric acid is 5 ≤ *x*_urate_ ≤ 10. The health range for the mass flow of all other intermediate metabolites is [0, 10]. To adjust the abnormal level of the disease-causing metabolite uric acid, we need to use some medication strategy, e.g. adjust the flux of some reaction by manipulating its enzyme activity. In our two-stage FBA method, such medication strategy is found by solving the medication model which minimizes the side effects. The computational result shows that 9 reactions have non-zero fluxes and there exist medication strategies that can adjust the level of uric acid to be normal (i.e. such that *x*_urate_ ∈ [5,10]) without causing side effects. To identify potential enzyme targets, we compare the fluxes of reactions obtained by the pathologic model and the medication model and find that the fluxes of 10 reactions are lower in the medication state than in the pathologic state. The enzymes catalyzing these reactions are xanthine oxidase, phosphoribosylpy-rophosphate synthetase, AMP deaminase, hypoxanthine phosphoribosyltransferase, adenine deaminase, IMP dehydrogenase, 5’-nucleotidase, and purine nucleosidase. In principle, adjusting the concentration of any of these enzymes can achieve the production reduction of uric acid and thus may act as potential drug targets. Xanthine oxidase, AMP deaminase, and 5’-nucleotidase are also identified as drug targets in [7]. According to our strategy for identifying potential drug targets, phosphoribosylpy-rophosphate synthetase, which is located in the source part of the hyperuricemia metabolic pathway and catalyzes the synthesis of PRPP, is a good choice since manipulating its concentration will naturally adjust the abnormally high fluxes of other downstream reactions, which eventually reduce the production of uric acid.

## Discussion

Classic FBA models only contain reaction fluxes without information of mass flows of metabolites. In this study, we define the mass flow of a metabolite as the weighted sum of the fluxes of all reactions that produce (or consume) this metabolite and incorporate it into the FBA model. Such an extended model allows us to track the change of mass flow of metabolites in the pathologic state and medication state. A similar metabolite-centric approach has been developed for metabolic network analysis and used in prediction of essential genes and discovery of antibacterials [39-41]. Such metabolite-centric approaches are expected to have many important applications as classic FBA models.

Our method finds the fluxes of reactions and the mass flows of metabolites in the medication state with the aim of minimizing side effects caused by medication. Since the side effects are closely related to the given healthy ranges, the single objective may make the LP model have multiple optimal solutions. A possible extension worth exploring is to use multiple objectives, such as minimizing side effects and maximizing biomass flux. In addition, Shlomi et al. developed a method for predicting metabolic disease biomarkers [42], which determines healthy/disease exchange intervals by optimizing the exchange reaction producing a boundary metabolite without or with including the reaction catalyzed by a dysfunctional enzyme. The boundary metabolite is predicted to be a biomarker of the dysfunctional reaction if the obtained disease exchange interval is significantly different from the healthy exchange interval. Similar ideas may be applied to drug target identification, in which a boundary enzyme crucial for the production of disease-causing compounds is predicted to be a potential drug target.

In this study, in addition to a simulated network, we also test our method on a true human purine metabolic pathway, which has been widely studied in the context of hyperuricemia. Enriched information is available about drugs and potential targets for this disease in the pathway. But complete quantitative information, such as the healthy ranges of metabolite flows and reaction fluxes in the pathologic state, still lacks. Matching the assumptions of our model with a true target network with complete quantitative information will be helpful for better validation as well as estimation of sensitivity and accuracy. However, so far there is no a database depositing such networks with detailed clinical data, though it is highly possible to have such data in hospitals from a clinical perspective. We believe our model and methods are practical once such data can be accessed publicly.

## Conclusions

Efficiently identifying drug targets with minimal side effects is one of major challenges in new drug development. High-throughput omics data provide unprecedent opportunities for drug target identification. Previous models for identifying drug targets either are not quantitative or do not consider side effects. In this paper, we develop a quantitative method based on flux balance analysis (FBA) to identify drug targets in metabolic networks. The method involves two linear programming (LP) models to find the steady optimal fluxes of reactions and the mass flows of metabolites in both pathologic state and medication state, meanwhile taking the side effects of drug action into account. The computational results on an illustrative simulated metabolic network and a hyperuricemia-related purine metabolic pathway show that the drug target identification problem can be solved effectively by the proposed two-stage FBA method.

## Authors contributions

ZL conceived the research and designed the models. ZL and RSW designed and performed the experiments. ZL, RSW and XSZ analyzed the data. All authors wrote, read and approved the paper.

## Competing interests

The authors declare that they have no competing interests.

## Supplementary Material

Additional file 1 **Reactions in the hyperuricemia metabolic pathway** This file contains the list of reactions in the hyperuricemia metabolic pathway with reaction IDs and Enzyme IDs. Click here for file

Additional file 2 **Compounds in the hyperuricemia metabolic pathway** This file contains the list of compounds in the hyperuricemia metabolic pathway. Click here for file

Additional file 3 **Enzymes in the hyperuricemia metabolic pathway** This file contains the list of enzymes in the hyperuricemia metabolic pathway. Click here for file
